# Effectiveness of Text Message Outreach to Promote Enrollment in a Government Food Assistance Program: Pilot Randomized Trial

**DOI:** 10.2196/78907

**Published:** 2025-10-31

**Authors:** Emily M Melnick, Francesco Acciai, Nicole Vaudrin O’Reilly, Mindy Jossefides, A Bea Ronan, Punam Ohri-Vachaspati

**Affiliations:** 1 College of Health Solutions, Arizona State University, 425 N 5th St, Phoenix, AZ, United States, 1 (480) 965-2100; 2 Department of Psychology, University of Denver, Denver, CO, United States; 3 Inter Tribal Council of Arizona, Phoenix, AZ, United States

**Keywords:** United States, WIC, SNAP, text messaging, intervention, women, infants, and children, Supplemental Nutrition Assistance Program

## Abstract

Findings from a pilot randomized trial of 916 households indicated that outreach text messages were not an effective strategy to increase enrollment in the United States Special Supplemental Nutrition Program for Women, Infants, and Children (WIC) among eligible households in Arizona.

## Introduction

The Special Supplemental Nutrition Program for Women, Infants, and Children (WIC) provides nutritious food packages, nutrition education, breastfeeding support, and health care referrals for pregnant and postpartum women and children aged 0‐4 years from low-income households in the United States [1]. WIC participation improves dietary and health outcomes and reduces health disparities [2]. However, many eligible households do not participate. Among those eligible for both WIC and the Supplemental Nutrition Assistance Program (SNAP) [3], another US food assistance program, about half participate in only SNAP and miss the additional benefits WIC provides [4]. In response, US agencies have called for outreach efforts to boost enrollment among SNAP-participating households eligible but not participating in WIC [5]. Increasing WIC participation is particularly important among American Indians who face higher rates of food insecurity [6], alongside declining WIC participation [7].

Text message outreach may offer a low-cost, scalable approach to encourage WIC enrollment among eligible SNAP-participating households [2]. No studies have evaluated impacts of such outreach in American Indian populations. This study evaluated a pilot randomized text message intervention aimed at increasing enrollment in the Inter Tribal Council of Arizona, Inc. (ITCA) WIC program. We hypothesized that households receiving persuasive outreach texts would be more likely to enroll in ITCA WIC than those who did not.

## Methods

### Study Overview

We conducted a pilot randomized trial among likely-WIC-eligible SNAP households in the ITCA WIC service area, including urban areas and tribal lands. The Arizona Department of Economic Security, which administers SNAP, provided the target population list. After eligibility screening, households were randomized in a 1:1 ratio to intervention and delayed control groups using a computerized random number generator. Participants were blinded to trial arm allocation, while evaluators were not. The study adhered to CONSORT (Consolidated Standards of Reporting Trials) guidelines (Checklist 1).

In February 2024, intervention households were sent a persuasive outreach text message via Teletask (Fair Oaks) that included the message, “Want healthy foods for your family? WIC is just a click away. With SNAP, you may already qualify.” The text message was developed by the study team in partnership with a communication specialist then field-tested with ITCA WIC local agency staff and low-income adults to assess persuasiveness and relevance for the target audience. Control households did not receive any texts during the 3-month (February-April 2024) evaluation period. To assess intervention effects, we conducted a per-protocol analysis, excluding 177 households in the intervention group that did not successfully receive the text message. Excluded households were demographically similar to those that successfully received the message, which, in turn, were similar to the delayed control group.

All analyses were run in Stata 16 (StataCorp). We used the command “proportion*”* to obtain group-specific enrollment proportions, estimated standard errors, and logit-transformed confidence intervals. A Pearson *χ*^2^ test (*α*=.05, two-tailed) compared enrollment rates across groups. The final analytical sample included 916 likely-WIC-eligible households with no missing data.

### Ethical Considerations

The intervention was implemented by ITCA WIC using data shared through an agreement with the Arizona Department of Economic Security. Because the text messages were sent as a part of programmatic improvement efforts and data shared were per a data sharing agreement protocol, consent to participate in this study was not required. All data were de-identified prior to analysis. Households did not receive compensation. The Arizona State University Institutional Review Board approved study protocols.

## Results

**Figure 1.**
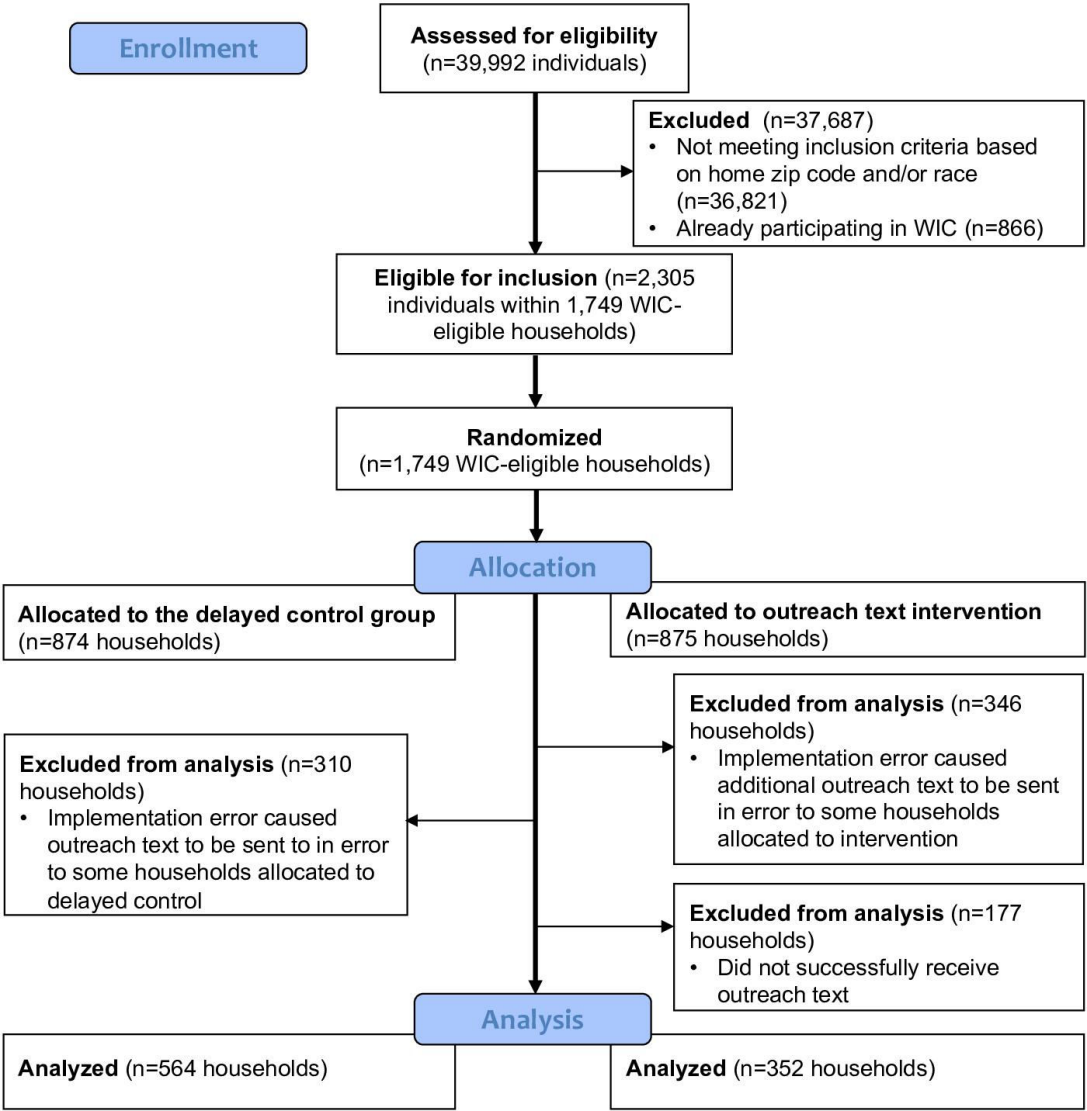
CONSORT flow diagram.

A CONSORT diagram (Figure 1) outlines intervention allocation and participant flow. Over half of the sample (516/916, 56.3%) identified as American Indian and 85.2% (780/916) lived in urban areas. During the 3-month follow-up, 54 of the 916 households (5.9%) enrolled in ITCA WIC. Enrollment was 4.3% (95% CI 2.6%‐7.0%) among households that received the text, while it was 6.9% (95% CI 5.1%‐9.3) among delayed controls. The difference was not statistically significant (*P*=.097, Pearson *χ*^2^ test).

## Discussion

This pilot study found no evidence that a text message increased WIC enrollment among likely-eligible SNAP households. This finding is consistent with results from pilot studies in Colorado, Montana, and Virginia [8]. While text interventions have improved outcomes such as retention of current WIC participants [9] and enrollment among WIC-eligible Medicaid participants [10], more intensive or multi-pronged approaches may be needed to increase WIC enrollment among eligible non-participating SNAP households. Notably, enrollment rates were low in both intervention and delayed control households. Possible reasons include children aging out of WIC eligibility before receiving the text and families already receiving services from another WIC agency in the state. Given this study’s focus on American Indian communities, future research should explore whether similar text-based interventions are effective in other populations and countries offering similar programs. We did not conduct a power calculation prior to the trial as we enrolled all eligible households; post-hoc analyses confirm the study was adequately powered to detect group differences. We also cannot rule out the possibility that intervention households shared messages with others. Future randomized trials should address the potential for cross-contamination between study arms. Trials with longer follow-up periods and those that test effects of multiple messages are also warranted.

## Supplementary material

10.2196/78907 Checklist 1 CONSORT checklist

## Abbreviations

ITCA: Inter Tribal Council of Arizona, Inc.
SNAP: Supplemental Nutrition Assistance Program
WIC: Special Supplemental Nutrition Program for Women, Infants, and Children
